# Decreased Urine N6-methyladenosine level is closely associated with the presence of diabetic nephropathy in type 2 diabetes mellitus

**DOI:** 10.3389/fendo.2022.986419

**Published:** 2022-09-27

**Authors:** Shu-jun Wan, Qiang Hua, Yu-jie Xing, Yi Cheng, Si-min Zhou, Yue Sun, Xin-ming Yao, Xiang-jian Meng, Jin-han Cheng, Han Wu, Qing Zhai, Yan Zhang, Xiang Kong, Kun Lv

**Affiliations:** ^1^ Central Laboratory, The first affiliated hospital of Wannan Medical College, Wuhu, China; ^2^ Key Laboratory of Non-coding RNA Transformation Research of Anhui Higher Education Institutes (Wannan Medical College), Wuhu, China; ^3^ Anhui Province Clinical Research Center for Critical Respiratory Medicine, Wuhu, China; ^4^ Department of Endocrinology, The first affiliated hospital of Wannan Medical College, Yijishan Hospital, Wuhu, China

**Keywords:** N6-methyladenosine, diabetic nephropathy, type 2 diabetes mellitus, biomarkers, urine

## Abstract

**Background:**

To investigate the dynamic changes of urine N6-methyladenosine (m6A) levels in patients with type 2 diabetes mellitus (T2DM) and diabetic nephropathy (DN) and evaluate the clinical significance.

**Methods:**

First, the levels of urine m6A were examined and compared among 62 patients with T2DM, 70 patients with DN, and 52 age- and gender-matched normal glucose tolerant subjects (NGT) by using a MethyIFIashTM Urine m6A Quantification Kit. Subsequently, we compared the concentrations of urine m6A between different stages of DN. Moreover, statistical analysis was performed to evaluate the association of urine m6A with DN.

**Results:**

The levels of m6A were significantly decreased in patients with DN [(16.10 ± 6.48) ng/ml], compared with NGT [(23.12 ± 7.52) ng/ml, *P* < 0.0001] and patients with T2DM [(20.39 ± 7.16) ng/ml, *P* < 0.0001]. Moreover, the concentrations of urine m6A were obviously reduced with the deterioration of DN. Pearson rank correlation and regression analyses revealed that m6A was significantly associated with DN (*P* < 0.05). The areas under the receiver operator characteristics curve (AUC) were 0.783 (95% CI, 0.699 – 0.867, *P* < 0.0001) for the DN and NGT groups, and 0.737 (95% CI, 0.639 – 0.835, *P* < 0.0001) for the macroalbuminuria and normoalbuminuria groups, and the optimal cutoff value for m6A to distinguish the DN from NGT and the macroalbuminuria from normoalbuminuria cases was 0.4687 (diagnostic sensitivity, 71%; diagnostic specificity, 76%) and 0.4494 (diagnostic sensitivity, 79%; diagnostic specificity, 66%), respectively.

**Conclusions:**

The levels of urine m6A are significantly decreased in patients with DN and change with the deterioration of DN, which could serve as a prospective biomarker for the diagnosis of DN.

## Introduction

Diabetic nephropathy (DN) is a progressive deterioration of renal function caused by long-term hyperglycemia in patients with diabetes, and it is known to be the primary cause of end-stage renal disease (1, 2). According to the latest epidemiological survey from the International Diabetes Federation, there are about 425 million patients with diabetes worldwide, among which about 20% – 40% will develop DN (2, 3). As DN is a chronic microvascular complication of diabetes, early diagnosis and prevention are of great importance to delay its occurrence and development. Oxidative stress, excessive production of advanced glycation end products, altered expression of cyclin-dependent kinases in cells, and increased activity of intracellular protein kinase C and sorbitol-aldose reductase are likely involved in the pathophysiological mechanisms underlying DN. More recently, some glomerular and tubular injury markers have been identified for the diagnosis of the DN pathological entities. In addition, assessment of urinary albumin excretion (UAE) and estimated glomerular filtration rate (eGFR) are currently suggested for DN annual screening (4). However, reliable early biomarkers for DN are still lacking and the specific molecular mechanisms underlying DN remain obscure.

Epigenetic changes in DNA, RNA, and histone modifications are associated with many diseases, including type 2 diabetes mellitus (T2DM) (5–7). In human and mouse cells, about 7,000 genes are modified with N6-methyladenosine (m6A) modifications, which are necessary for transcription, translation, and microRNA maturation (8, 9). It has been reported that m6A modifications change dynamically with disease progression (9–12), which indicates that m6A plays an important role in physiology and pathology and may become a potential biomarker for diseases. Moreover, m6A plays a certain role in maintaining the stability of glucose in rat adipocytes by promoting glucose oxidation (13); the levels of m6A in peripheral blood negatively correlate with the progression of T2DM (14); and a high glucose environment in patients with T2DM could lead to the decrease of m6A content and thus affect the glucose and lipid metabolism processes (14, 15). Therefore, m6A modification is closely related to the occurrence and development of T2DM. Moreover, m6A RNA modification may be involved in the pathogenesis and progression of diabetic retinopathy (16). METTL14 plays an important role in DN through m6A modification of α-Klotho (17). WTAP promotes m6A methylation of NLRP3 mRNA, which further induces cell pyroptosis and inflammation in DN (18). However, the dynamic changes of m6A levels in DN are still unclear.

Therefore, in the present study, we examined and compared the urine m6A levels among patients with noncomplicated T2DM, patients with DN, and normal glucose tolerant subjects (NGT), and we assessed possible correlations between the m6A content and clinical biochemical parameters. In addition, we explored m6A levels in different development stages of DN and evaluated the potential of the m6A as a diagnostic biomarker for DN.

## Methods

### Sample collection and study design

G. power3.1.9.7 software was adopted to calculate the sample size. Urine samples of 62 patients with noncomplicated T2DM, 70 patients with DN, and 52 NGT were collected from the Department of Clinical Laboratory, Yijishan Hospital, from January 2020 to October 2020. The diagnosis of T2DM was established in accordance with the 1999 World Health Organization diagnostic criteria as follows: fasting plasma glucose ≥ 7.0 mmol/L (126 mg/dl), and/or 2-h glucose ≥ 11.1 mmol/L (200 mg/dl) in the 75-g OGTT, and/or HbA1c ≥ 6.5% (19). The clinical diagnosis of DN was based on spot urinary albumin to creatinine ratio (UACR) of > 30 mg/g more than twice in 3 months in patients with T2DM (20). The study protocol was approved by the Scientific Research and New Technology Ethics Committee of Wannan Medical College, Yijishan Hospital (No. 202022). Informed consent was obtained from all of the participants before the study.

The exclusion criteria included the presence of primary hypertension, hepatic injury, malignant tumor, lupus erythematosus, other kidney diseases (such as nephrotic syndrome, nephritis, and renal insufficiency), other endocrine diseases (such as hyperthyroidism, hypothyroidism, pheochromocytoma, and hypercortisolism), and the use of nephrotoxic drugs. Urine samples from participants with heavy ketonuria were excluded from this study. The samples were collected in deenzyme tubes and then stored at −80°C until use.

To further explore the relationship between m6A and DN, the patients were divided into three groups according to their UACR: the normoalbuminuria group (UACR < 30 mg/g), microalbuminuria group (30 mg/g < UACR < 300 mg/g), and macroalbuminuria group (UACR > 300 mg/g) (21).

### Determination of serum biochemical parameters

Serum glucose levels and other clinical biochemical parameters, including serum creatinine (Cr), blood urea nitrogen (BUN), total cholesterol (TC), and triglycerides (TG), were examined by an auto-analyzer (Hitachi 7600, Hitachi High-Technologies Inc., Tokyo, Japan). C-peptide concentration was measured using commercial reagents on an ADVIA Centaur XP Immunoassay System (SIEMENS Inc., Munich, Germany). Glycosylated hemoglobin A_1C_ (HbA_1c_) was assessed using commercial reagents on an HA-8180 auto-analyzer (Arkrayha Inc., Tokyo, Japan). Proteinuria, urinary albumin, and creatinine were detected on an Hitachi 7600 analyzer (Hitachi High-Technologies Inc., Tokyo, Japan).

### Urine m6A quantification

The levels of m6A in total RNA and DNA were measured by using a MethyIFIashTM Urine m6A Quantification Kit (Epigentek Group Inc., USA). Briefly, 5 μl of urine was added to assay wells covered with binding solution. Then, capture antibody solution, detection antibody solution, and enhancer solution were added to assay holes with diluted concentration in accordance with the manufacturer’s instructions. Finally, development and stop solution were added to produce a color reaction. The absorbance of each well was measured at a wavelength of 450 nm, and the m6A levels were calculated based on the standard curve.

### Statistical analysis

Statistical analysis was performed with SPSS 22.0 and GraphPad Prism version 8.0. The levels of m6A and other continuous variables were presented as the mean ± SD. The differences in the m6A concentrations and other variables among groups were analyzed by two-sided χ^2^ test, one-way ANOVA followed by least-significant difference (LSD) test, or nonparametric Mann–Whitney *U* test. Pearson rank correlation analysis was used to measure the strength of association between two variables. Logistic regression and receiver operating characteristic curve (ROC) analysis were also conducted to evaluate the influence and diagnostic value of urine m6A for DN. A *P* value < 0.05 was considered to be statistically significant.

## Results

### Clinical characteristics of the participants

The clinical features of the participants including 62 patients with T2DM, 70 patients with DN, and 52 NGT are listed in **Table 1**. There were no significant differences in sex and mean age among the three groups. Patients with DN showed higher body mass index (BMI), systolic blood pressure (SBP), diastolic blood pressure (DBP), fasting blood glucose (FBG), TG, and BUN than NGT (*P* < 0.05). Furthermore, hypertension percentage, SBP, antihypertensives drug treatment, use of angiotensin-receptor blocker/angiotensin-converting enzyme inhibitors (ARB/ACEI), other antihypertensives drugs, and insulin, and levels of TG, Cr, and BUN were significantly elevated, while eGFR was obviously decreased in patients with DN compared with patients with T2DM (*P* < 0.05).

**Table 1:** Demographic and clinical features of the NGT, T2DM and DN patients.

	NGT	T2DM	*P* ^b^	DN	*P* ^b^	*P* ^c^
**n**	52	62		70		
**Sex-no. (%)**			0.182^d^		0.056^d^	0.569^d^
**Male**	30 (57.69)	42 (67.74)		50 (71.43)		
**Female**	22 (42.31)	20 (32.26)		20 (28.57)		
**BMI (kg/m^2^)**	23.89 ± 2.83	24.64 ± 4.43	0.638	25.71 ± 4.27	0.024	0.449
**Age (years)**	48.81 ± 6.49	48.40 ± 10.57	0.275	49.76 ± 9.30	0.112	0.500
**Duration of diabetes (years)**		8.16 ± 6.24		9.63 ± 5.38		0.103
**Hypertension no (%)**		12 (19.36)		34 (48.57)		<0.0001^d^
**SBP (mmHg)**	127.37 ± 11.16	132.08 ± 17.27	0.236	143.59 ± 23.66	<0.0001	0.002
**DBP (mmHg)**	76.55 ± 8.40	82.23 ± 8.66	0.002	84.33 ± 13.36	<0.0001	0.627
**Smoking status-no. (%)**			0.820^d^		0.136^d^	0.178^d^
**Ever and current**	6 (11.54)	8 (12.90)		15 (21.43)		
**Never**	46 (88.46)	54 (87.10)		55 (78.57)		
**Alcohol consumption-no. (%)**			0.235^d^		0.027^d^	0.207^d^
**Ever and current**	1 (1.92)	4 (6.45)		9 (12.86)		
**Never**	51 (98.08)	58 (93.55)		61 (87.14)		
**Antihypertensives drug treatment-no. (%)**						
**ARB/ACEI**		7 (11.29)		18 (25.71)		0.006^d^
**Others**		7 (11.29)		20 (28.57)		0.001^d^
**Never**		52 (83.87)		43 (61.43)		<0.0001^d^
**Hypoglycemic treatment no. (%)**						
**Insulin**		13 (20.97)		25 (35.71)		0.019^d^
**Hypoglycemic drugs**		35 (56.45)		36 (51.43)		0.510^d^
**Never**		22 (35.48)		18 (25.71)		0.185^d^
**FBG (mmol/l)**	4.99 ± 0.35	9.19 ± 3.37	<0.0001	9.61 ± 3.61	<0.0001	0.574
**TC (mmol/l)**	4.35 ± 0.50	4.27 ± 1.21	0.092	4.93 ± 3.29	0.480	0.077
**TG (mmol/l)**	1.38 ± 0.52	2.10 ± 3.01	0.103	2.75 ± 1.78	<0.0001	<0.0001
**Cr (μmol/l)**	76.94 ± 25.92	69.18 ± 25.04	<0.0001	93.25 ± 44.66	0.156	0.001
**Bun (mmol/l)**	4.76 ± 1.12	5.40 ± 1.70	0.018	6.77 ± 3.25	<0.0001	0.004
**eGFR (ml/min/1.73m^2^)**		118.46 ± 54.55		93.24 ± 60.30		0.010
**Diabetic complications no. (%)**						
**Diabetic retinopathy**		5 (8.06)		8 (11.43)		0.382^d^
**Diabetic peripheral neuropathy**		10 (16.13)		11 (15.71)		0.932^d^

^a^Data are mean ± SD or number (%). ^b^Compared with control group. ^c^Compared between the two case groups. ^d^ Two-sided χ^2^ test. NGT, normal glucose tolerant subjects; T2DM, type 2 diabetes mellitus; DN, diabetic nephropathy; BMI, body mass index; SBP, systolic blood pressure; DBP, diastolic blood pressure; FBG, fasting blood glucose; TC, total cholesterol; TG, triglycerides; Cr, creatinine; BUN, blood urea nitrogen; eGFR, estimated glomerular filtration rate; ARB, angiotensin-receptor blocker; ACEI, angiotensin-converting enzyme inhibitors.

### Urine levels of m6A are decreased in patients with DN

**Figure 1:**
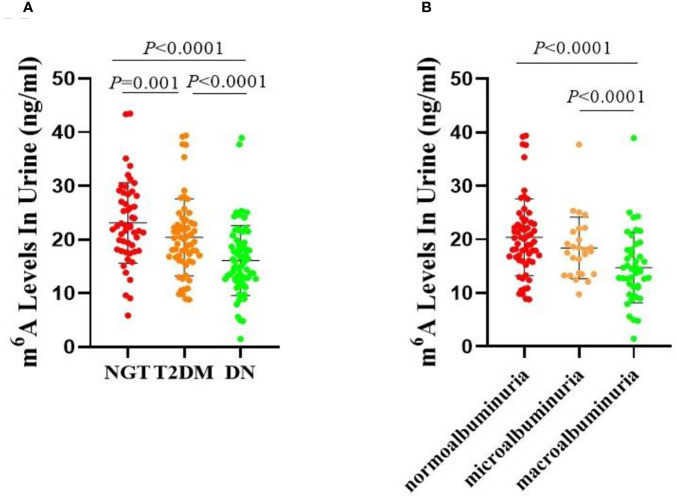
Urine levels of m6A in patients. **(A)** Absolute concentrations of the urine m6A in the NGT, T2DM and DN groups. **(B)** Absolute concentrations of the urine m6A in the normoalbuminuria, microalbuminuria and macroalbuminuria groups.

We first calculated the urine levels of m6A in the NGT, T2DM, and DN groups. The concentration of m6A was obviously lower in the DN group compared with the NGT (*P* < 0.0001) and T2DM (*P* < 0.0001) groups (**Figure 1A**). Furthermore, a significant difference was also observed between the T2DM and NGT groups (*P =* 0.001) (**Figure 1A**). Taken together, these data demonstrated that the urine m6A levels were significantly decreased in patients with DN and patients with T2DM, and the decrease of m6A was more obvious in the DN group.

### Urine m6A levels by age distribution

**Figure 2:**
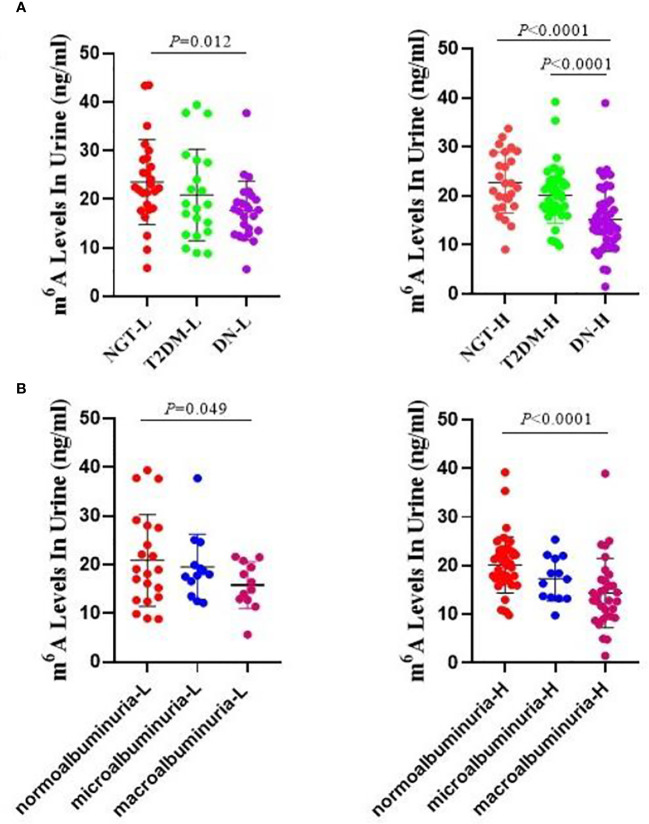
Urine m6A levels by age distribution. **(A)** Absolute concentrations of the urine m6A in the NGT-L, NGT-H, T2DM-L, T2DM-H, DN-L and DN-H groups. **(B)** Absolute concentrations of the urine m6A in the normoalbuminuria-L, normoalbuminuria-H, microalbuminuria-L, microalbuminuria-H, macroalbuminuria-L and macroalbuminuria-H groups.

As age can be an important factor in the development of diabetic microvascular complications, we investigated the m6A levels in different age groups. As the average age of all of the participants was 48.99 years, we set 49 years as the critical point for age division, and we evaluated the following subgroups: the NGT low-age group (NGT-L), NGT high-age group (NGT-H), T2DM low-age group (T2DM-L), T2DM high-age group (T2DM-H), DN low-age group (DN-L), and DN high-age group (DN-H). We found that the levels of m6A were significantly lower in the DN-L and DN-H groups than in the NGT-L (*P* = 0.012), NGT-H (*P* < 0.0001), and T2DM-H (*P* < 0.0001) groups. However, there were no significant differences between the NGT-L and NGT-H, T2DM-L and T2DM-H, and DN-L and DN-H groups (**Figure 2A**). When analyzing normoalbuminuria low-age group (normoalbuminuria-L), normoalbuminuria high-age group (normoalbuminuria-H), microalbuminuria low-age group (microalbuminuria-L), microalbuminuria high-age group (microalbuminuria-H), macroalbuminuria low-age group (macroalbuminuria-L), and macroalbuminuria high-age group (macroalbuminuria-H), we found that m6A was significantly lower in the macroalbuminuria-L and macroalbuminuria-H groups than in normoalbuminuria-L (*P* = 0.049) and normoalbuminuria-H (*P* < 0.0001) groups. However, there were no significant differences in the normoalbuminuria-L vs normoalbuminuria-H, microalbuminuria-L vs microalbuminuria-H, and macroalbuminuria-L vs macroalbuminuria-H (**Figure 2B**). We suspect that the slight differences between the low- and high-age groups were due to the smaller sample size in the lower age group.

### Changes in urine m6A in the different stages of DN

The clinical characteristics of the three groups are listed in **Table 2**. The duration of diabetes, hypertension percentage, SBP, antihypertensive drug treatment, ARB/ACEI, other antihypertensive drugs, insulin usage, fasting plasma C-peptide (FC-P), TC, TG, Cr, BUN, 24-h urine protein quantitative (24-h Upro), UACR levels, and diabetic retinopathy rate were significantly higher, while eGFR was obviously lower in the macroalbuminuria group compared with the normoalbuminuria group (**Table 2**). Moreover, there were marked differences in insulin usage, 24-h Upro, UACR, eGFR, and diabetic retinopathy rate between the microalbuminuria and macroalbuminuria groups (**Table 2**).

**Table 2:** Demographic and clinical features of the normoalbuminuria, microalbuminuria and macroalbuminuria.

	normoalbuminuria	microalbuminuria	*P* ^b^	macroalbuminuria	*P* ^b^	*P* ^c^
**n**	62	26		44		
**Sex-no. (%)**			0.036^d^		0.592^d^	0.016^d^
**Male**	42 (67.74)	22 (84.62)		28 (63.64)		
**Female**	20 (32.26)	4 (15.38)		16 (36.36)		
**Age (years)**	48.40 ± 10.57	47.19 ± 10.49	0.433	51.27 ± 8.28	0.134	0.060
**BMI (kg/m^2^)**	24.64 ± 4.43	25.92 ± 4.06	0.214	25.55 ± 4.48	0.328	0.745
**Duration of diabetes (years)**	8.16 ± 6.24	7.95 ± 5.61	0.988	10.46 ± 5.13	0.026	0.101
**Hypertension no. (%)**	12 (19.36)	14 (53.85)	<0.0001^d^	20 (45.46)	<0.0001^d^	0.434^d^
**SBP (mmHg)**	132.08 ± 17.27	138.85 ± 21.14	0.150	146.39 ± 24.84	0.002	0.200
**DBP (mmHg)**	82.23 ± 8.66	85.38 ± 11.15	0.497	83.70 ± 14.60	0.909	0.931
**Smoking status-no. (%)**			0.424^d^		0.163^d^	0.716^d^
**Ever and current**	8 (12.90)	5 (19.23)		10 (22.73)		
**Never**	54 (87.10)	21 (80.77)		34 (77.27)		
**Alcohol consumption-no. (%)**			0.409^d^		0.200^d^	0.794^d^
**Ever and current**	4 (6.45)	3 (11.54)		6 (13.64)		
**Never**	58 (93.55)	23 (88.46)		38 (86.36)		
**Antihypertensive drug treatment-no. (%)**						
**ARB/ACEI**	7 (11.29)	6 (23.08)	0.066^d^	12 (27.27)	0.006^d^	0.623^d^
**Others**	7 (11.29)	7 (26.92)	0.019^d^	13 (29.55)	0.002^d^	0.769^d^
**Never**	52 (83.87)	18 (69.23)	0.050^d^	25 (56.82)	<0.0001^d^	0.210^d^
**Hypoglycemic treatment-no. (%) **						
**Insulin**	13 (20.97)	4 (15.38)	0.429^d^	21 (47.73)	<0.0001^d^	0.001^d^
**Hypoglycemic drugs**	35 (56.45)	14 (53.85)	0.798^d^	22 (50.00)	0.452^d^	0.722^d^
**Never**	22 (35.48)	9 (34.61)	0.932^d^	9 (20.45)	0.072^d^	0.156^d^
**FBG (mmol/l)**	9.19 ± 3.37	9.54 ± 2.86	0.667	9.65 ± 4.02	0.514	0.907
**2-h PBG (mmol/l)**	13.74 ± 5.48	15.63 ± 5.72	0.199	14.88 ± 6.69	0.363	0.636
**FC-P**	1.15 ± 0.65	1.57 ± 1.14	0.214	1.98 ± 1.79	0.014	0.419
**2-h C-P**	2.76 ± 2.05	2.83 ± 1.62	0.528	3.31 ± 2.64	0.380	0.824
**HbA_1c_ (%)**	9.45 ± 2.65	9.55 ± 2.17	0.685	9.09 ± 2.23	0.611	0.433
**TC (mmol/l)**	4.27 ± 1.21	4.21 ± 1.09	0.835	5.34 ± 4.00	0.009	0.051
**TG (mmol/l)**	2.10 ± 3.01	2.74 ± 1.76	0.012	2.76 ± 1.82	<0.0001	0.920
**Cr (μmol/l)**	69.18 ± 25.04	82.32 ± 38.22	0.136	99.50 ± 47.26	<0.0001	0.110
**Bun (mmol/l)**	5.40 ± 1.70	6.23 ± 2.53	0.081	7.08 ± 3.59	0.002	0.235
**24-h Upro (mg)**	0.05 ± 0.03	0.34 ± 0.20	<0.0001	1.79 ± 1.68	<0.0001	<0.0001
**UACR (mg/g)**	9.19 ± 6.03	149.00 ± 80.15	<0.0001	1906.90± 1941.06	<0.0001	<0.0001
**eGFR (ml/min/1.73m^2^)**	118.46 ± 54.55	114.32 ± 65.70	0.876	80.78 ± 53.84	<0.0001	0.023
**Diabetic complications no. (%)**						
**Diabetic retinopathy**	5 (8.06)	0 (0)	0.037	8 (11.43)	0.038^d^	0.002^d^
**Diabetic peripheral neuropathy**	10 (16.13)	6 (23.08)	0.321	5 (11.36)	0.357^d^	0.091^d^

^a^Data are mean ± SD or number (%). ^b^Compared with normoalbuminuria group. ^c^Compared between the two case groups. ^d^ Two-sided χ^2^ test. BMI, body mass index; SBP, systolic blood pressure; DBP, diastolic blood pressure; FBG, fasting blood glucose; 2-h PBG, two-hours postprandial blood glucose; FC-P, fasting plasma C-peptide; 2-h C-P, two-hours postprandial C-peptide; HbA_1c_, hemoglobin A_1c_; TC, total cholesterol; TG, triglycerides; Cr, creatinine; BUN, blood urea nitrogen; 24-h Upro, 24-hours urine protein quantitative; UACR, urinary albumin creatinine ratio; eGFR, estimated glomerular filtration rate; ARB, angiotensin-receptor blocker; ACEI, angiotensin-converting enzyme inhibitors.

As shown in **Figure 1B**, m6A contents were lower in both the microalbuminuria and macroalbuminuria groups than in the normoalbuminuria group, and the difference between the macroalbuminuria and normoalbuminuria groups was statistically significant (*P* < 0.0001). Moreover, remarkable reduction was observed between the microalbuminuria and macroalbuminuria groups (*P* < 0.0001, **Figure 1B**). Taken together, the results showed that m6A levels were decreased with the deterioration of DN.

### Relationships of urine m6A with biochemical parameters

Next, we investigated whether urine m6A levels were associated with serological biochemical parameters. We evaluated associations of the clinical features with m6A levels using Pearson rank correlation analysis in all of the enrolled subjects. As demonstrated in **Table 3**, urine m6A levels showed significantly negative relationships with hypertension percentage, antihypertensive drugs (except for ARB/ACEI), FC-P, BUN, and 24-h Upro, and a positive relationship with eGFR (*P* < 0.05 or 0.01). These data suggest that the downregulation of urine m6A may be involved in the pathogenesis of DN.

**Table 3:** Pearson rank correlations between urinary m6A and sera and urine indices in all the studied samples.

	m6A	
Varibles	r	*P*
**Sex**	-0.120	0.105
**Age**	-0.109	0.144
**BMI**	-0.037	0.630
**SBP**	-0.050	0.503
**DBP**	-0.032	0.664
**Smoking status**	-0.120	0.106
**Alcohol consumption**	-0.026	0.731
**Duration of diabetes**	0.047	0.635
**Hypertension percentage**	-0.181	0.039*
**Antihypertensive drug treatment**	-0.155	0.078
**ARB/ACEI**	-0.054	0.537
**Other antihypertensive drugs**	-0.172	0.049*
**Hypoglycemic treatment**	0.015	0.867
**Insulin**	-0.102	0.246
**Hypoglycemic drugs**	0.094	0.287
**FBG**	-0.108	0.148
**2-h PBG**	-0.019	0.840
**FC-P**	-0.193	0.028*
**2-h C-P**	-0.120	0.174
**HbA_1c_**	0.123	0.181
**TC**	-0.129	0.083
**TG**	0.0001	0.998
**Cr**	0.0001	0.996
**BUN**	-0.167	0.025*
**24-h Upro**	-0.233	0.008**
**eGFR**	0.253	0.004**
**Diabetic retinopathy**	-0.125	0.156
**Diabetic peripheral neuropathy**	0.065	0.462

FBG, fasting blood glucose; 2-h PBG, two-hours postprandial blood glucose; FC-P, fasting plasma C-peptide; 2-h C-P, two-hours postprandial C-peptide; HbA_1c_, hemoglobin A_1c_; TC, total cholesterol; TG, triglycerides; Cr, creatinine; BUN, blood urea nitrogen; 24-h Upro, 24-hours urine protein quantitative; eGFR, estimated glomerular filtration rate; ARB, angiotensin-receptor blocker; ACEI, angiotensin-converting enzyme inhibitors. *P < 0.05, **P < 0.01.

### Decreased urine levels of m6A are closely associated with the presence of DN

To weigh the clinical usefulness of the decreased urine m6A in patients with DN, we performed a forward stepwise binary logistic regression in two models. Model 1 consisted of the NGT, T2DM, and DN groups, while model 2 consisted of the normoalbuminuria, microalbuminuria, and macroalbuminuria groups. In model 1, regardless of whether the NGT or T2DM group was used as the reference category, urine m6A was independently associated with DN, with the odds ratios (ORs) of 0.859 (95% CI, 0.803 – 0.919*, P <* 0.0001) and 0.897 (95% CI, 0.857 – 0.939, *P <* 0.0001), respectively (**Table 4**). To further assess the association of urine m6A with different degrees of DN, we analyzed model 2. We found that m6A was independently associated with macroalbuminuria, when the normoalbuminuria and microalbuminuria groups were treated as reference categories (OR = 0.873, 95% CI, 0.810 – 0.941, *P* < 0.0001; OR = 0.909, 95% CI, 0.834 – 0.992, *P* = 0.033, respectively) (**Table 4**). These results suggest that the decrease of urine m6A is a potential independent risk factor for the presence of DN. Furthermore, multivariate logistic regression analysis was subsequently performed to confirm the correlation of urine m6A with DN. After adjusting for BMI, SBP, DBP, FBG, FC-P, TC, TG, Cr, BUN, eGFR, hypertension percentage, use of ARB/ACEI, other antihypertensive drugs, no antihypertensive drug treatment, duration of diabetes, and insulin, we observed that urine m6A still remained independently associated with DN in model 1 and with macroalbuminuria in model 2 (**Table 4**).

**Table 4:** Univariate and multivariate logistic regression analyses of urinary m6A for DN.

M6A			Univariate		Multivariate	
			OR (95%CI)	*P*	OR (95%CI)	*P*
**Model 1**	**A**	**T2DM**	0.950 (0.901~1.001)	0.054		
		**DN**	0.859 (0.803~0.919)	<0.0001	0.657 (0.486~0.888)	0.006
	**B**	**DN**	0.897 (0.857~0.939)	<0.0001	0.898 (0.846~0.954)	<0.0001
**Model 2**	**a**	**microalbuminuria**	0.953 (0.884~1.027)	0.210		
		**macroalbuminuria**	0.873 (0.810~0.941)	<0.0001	0.885 (0.802~0.977)	0.015
	**b**	**macroalbuminuria**	0.909 (0.834~0.992)	0.033		

Model 1 were consisted of NGT, T2DM and DN groups. Model 2 were consisted of normoalbuminuria, microalbuminuria and macroalbuminuria groups. A: the reference category was the NGT group. B: the reference category was the T2DM group. a: the reference category was the normoalbuminuria group. b: the reference category was the microalbuminuria group.

### Diagnostic utility of urine m6A in DN

**Figure 3:**
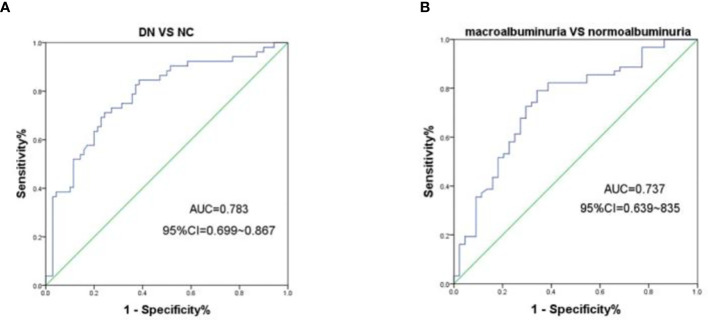
ROC curves for the capacity of the urine m6A to differentiate the DN from NGT or the macroalbuminuria from normoalbuminuria individuals. **(A)** ROC curves for the ability of the concentrations of urine m6A to differentiate the DN cases (n = 70) from NGT (n = 52). **(B)** ROC curves for the ability of the concentrations of urine m6A to differentiate the macroalbuminuria (n = 44) from normoalbuminuria (n = 62) cases.

To further evaluate the association of urine m6A with DN, we performed ROC analysis on m6A in all of the patients and controls enrolled in this study, and yielded an area under the receiver operator characteristics curve (AUC) of 0.783 (95% CI, 0.699 – 0.867, *P* < 0.0001) for the DN and NGT groups (**Figure 3A**). Moreover, we further evaluated the diagnostic value of urine m6A in patients and found that the AUC was 0.737 (95% CI, 0.639 – 0.835, *P* < 0.0001) for the macroalbuminuria and normoalbuminuria groups (**Figure 3B**). The optimal cutoff value for m6A to distinguish the DN from NGT and the macroalbuminuria from normoalbuminuria was 0.4687 (diagnostic sensitivity, 71%; diagnostic specificity, 76%) and 0.4494 (diagnostic sensitivity, 79%; diagnostic specificity, 66%), respectively. Taken together, the above results indicate that urine m6A has a high diagnostic value for DN.

## Discussion

Modification of m6A is widely involved in replication, transcription, and translation of genes in cells, and its abnormal expression is associated with various diseases (22, 23). As a highly conserved methylated modification, m6A levels in RNA are significantly reduced both in patients with T2DM and in rats (15). Relevant reports have shown that m6A modification could promote the expression of p53 mRNA and protein, thereby aggravating kidney injury in experiments of acute kidney injury *in vivo* and *in vitro* (24). However, dynamic changes of m6A in DN are largely unclear. It is well known that urine is a more direct indicator of pathological changes in the kidney than serum. Therefore, in the present study, we analyzed the m6A content in urine of patients with DN and found that the levels of m6A were obviously lower in patients with DN than in NGT and patients with T2DM. We further explored the changes in m6A levels between different development stages of DN, and found that the m6A level continued to reduce with the deterioration of DN, which was closely related to the presence of DN.

As DN is one of the chronic microvascular complications of T2DM, its incidence increases significantly with the continuous prolongation of the disease, and once DN occurs, it is difficult to reverse it. Therefore, early diagnosis and prevention are particularly important to reduce the prevalence and improve the prognosis of DN. Urinary protein and microalbumin are the most common monitoring indexes of DN internationally, and they have high clinical value in the evaluation of renal function (25). However, the above indicators cannot predict the occurrence of DN and they have a large individual variation, with about 28% of patients without proteinuria in the progression of DN (26, 27). Moreover, patients with DN often have hypertension, and routine proteinuria detection cannot accurately distinguish DN from hypertensive nephropathy (28). Although renal aspiration and tissue biopsy are currently the gold standard for the diagnosis of DN, they are invasive and limited by the operative skills and experience of the sampler; thus, they are difficult to popularize in clinical practice. In addition, GFR is estimated by serum creatinine, which is susceptible to various factors, and the results lack reliability (29). Therefore, the search for DN diagnostic markers with higher sensitivity and specificity and better clinical effect has become an urgent clinical need. Animal and clinical studies have shown that histone modification, post-transcriptional RNA regulation, and DNA methylation are involved in the occurrence and development of diabetic microvascular complications (30). Li et al. indicated that reduced histone H3-lysine9-dimethylation (H3K9me2), increased histone H3-lysine4 methylation (H3K4me1/3), and translocation of SET7/9 at the p21 promoter region could increase the expression of p21 in high glucose–treated mesangial cells (31). Furthermore, abnormal DNA methylation of TGF-β1 caused by excessive reactive oxygen species plays a key role in mesangial fibrosis in the progression of DN (32). These preliminary studies have indicated that epigenetic changes play an important role in the development of DN, which may bring new insights for novel biomarkers of DN.

M6A modification is the most common chemical modification of eukaryotic mRNA and prokaryotic DNA (9, 22). The levels of RNA m6A in peripheral blood of patients with gastric cancer are significantly higher than those of patients with benign gastric diseases and normal controls, and they continue to increase as the cancer grows and metastasizes (33). In addition, m6A is elevated in renal fibrosis induced by TGF-β1, and it participates in the MALAT1/miR-145/focal adhesion kinase signaling pathway, which affects the pathological process of chronic renal diseases (34). Therefore, m6A is worthy of further research as a potential diagnostic and monitoring indicator. In this study, the urine levels of m6A of patients with DN were significantly reduced, which is consistent with the decrease of m6A in the circulating blood of diabetes patients (15). Moreover, the levels of m6A decreased gradually with the development of DN, which was closely related to the pathological process of DN. Urine m6A negatively correlates with pancreas islet and renal function indexes, and it is a potential independent risk factor for DN, indicating that m6A is linked to the dysfunction of diabetic kidneys.

As the most common chemical modification of mRNA, m6A can affect maturation, transcription, localization, translation, and metabolism of RNA (35). DNA m6A modification is also common in human cells, and its reduced levels always promote tumorigenesis (36). In the present study, we detected levels of urine DNA and RNA m6A. Due to the limited detection methods, it is difficult to distinguish the origin of DN urine m6A from DNA or RNA, which should be explored in future studies. Furthermore, it should be pointed out that this was a single-center, small-sample experiment. In order to confirm that m6A could be used as a marker for early clinical diagnosis of DN, we need to carry out multicenter studies and expand the sample size.

In conclusion, in this study, we showed for the first time that m6A levels are significantly reduced in urine of patients with DN and decrease gradually with the deterioration of DN, which indicates that urine m6A is closely related to DN and has the potential to be an early diagnosis and monitoring biomarker for it.

## Data availability statement

The original contributions presented in the study are included in the article/supplementary material. Further inquiries can be directed to the corresponding authors.

## Ethics statement

The studies involving human participants were reviewed and approved by Scientific Research and New Technology Ethics Committee of Wannan Medical College, Yijishan Hospital. The patients/participants provided their written informed consent to participate in this study.

## Author contributions

Conception or design: S-JW, XK, and KL. Acquisition, analysis, or interpretation of data: S-JW, Y-JX, S-MZ, QH, YC, YS, X-MY, X-JM, J-HC, HW, QZ, and YZ. Drafting the work or revising: S-JW. Final approval of the manuscript: QH, XK, and KL. All authors contributed to the article and approved the submitted version.

## Funding

This study was supported by the grants from the National Natural Science Foundation of China (81970699 and 82072370), Key University Science Research Project of Anhui Province (KJ2020A0594), Key Laboratory of Non-coding RNA Transformation Research of Anhui Higher Education Institution (Wannan Medical College) (RNA202007), and ‘Peak’Training Program for Scientific Research of Yijishan Hospital, Wannan Medical College (GF2019J04).

## Conflict of interest

The authors declare that the research was conducted in the absence of any commercial or financial relationships that could be construed as a potential conflict of interest.

## Publisher’s note

All claims expressed in this article are solely those of the authors and do not necessarily represent those of their affiliated organizations, or those of the publisher, the editors and the reviewers. Any product that may be evaluated in this article, or claim that may be made by its manufacturer, is not guaranteed or endorsed by the publisher.
